# Structural and Morphological Features of Disperse Alumina Synthesized Using Aluminum Nitrate Nonahydrate

**DOI:** 10.1186/s11671-016-1366-0

**Published:** 2016-03-22

**Authors:** Ivan F. Myronyuk, Volodymyr I. Mandzyuk, Volodymyr M. Sachko, Volodymyr M. Gun’ko

**Affiliations:** Vasyl Stefanyk Precarpathian National University, 57 Shevchenko Street, 76018 Ivano-Frankivsk, Ukraine; Chuiko Institute of Surface Chemistry, 17 General Naumov Street, 03164 Kyiv, Ukraine

**Keywords:** Aluminum nitrate nonahydrate, Aluminum hydroxide, Alumina, Nanoparticle morphology, Aggregate texture, 61.66.Fn, 64.70.K, 74.62.Bf

## Abstract

Transformation of Al(NO_3_)_3_∙9H_2_O (upon heating in the range of 20–1200 °C) into blends of amorphous and crystalline boehmite (210–525 °C), amorphous alumina and crystalline γ-Al_2_O_3_ (850 °C), and crystalline α-Al_2_O_3_ (1100 °C) was analyzed using X-ray diffraction (XRD), high-resolution transmission electron microscope (HRTEM), infrared (IR) spectroscopy, thermogravimetry, and low-temperature nitrogen adsorption. Boehmite consists of nanoparticles of 6–10 nm in diameter, and part of them has crystalline structure observed in HRTEM images, despite they are XRD amorphous. The nanoglobules are surrounded by amorphous aluminum hydroxide with chains of –AlO(H)–O–AlO(H)– of 1–5 nm in length. Heating of samples at 350–525 °C gives mesoporous aluminum hydroxide with a relatively narrow pore size distribution. An increase in calcination temperature to 850 °C decreases the porosity of alumina composed of amorphous and crystalline (γ-Al_2_O_3_) phases. Calcination at 1100 °C gives α-Al_2_O_3_ with strongly decreased porosity of aggregates.

**Electronic supplementary material:**

The online version of this article (doi:10.1186/s11671-016-1366-0) contains supplementary material, which is available to authorized users.

## Background

Alumina in various modifications, individual crystalline (see Additional file 1: Figure S1, Table S1) and amorphous or in complex oxides such as clays and zeolites, is widely used in industry, scientific equipment, medicine, etc. [1–6]. Among different phases of alumina, corundum (α-Al_2_O_3_) is characterized by very high hardness (equal to 9 according to Mohs scale), high melting point (2044 °C), and different colors when impurities are present (sapphires, etc.), i.e., corundum is of interest for various applications. In industrial production of α-Al_2_O_3_, natural minerals such as bauxite, nepheline, and alunite are used in various processes [1–6]. Natural precursors are characterized by the presence of a variety of admixtures that make difficult production of pure aluminas. The use of pure precursors such as AlCl_3_ in flame synthesis of alumina results in the formation of nanoparticles with a blend of different crystalline and amorphous phases depending on the flame temperature and temperature gradient. If the flame temperature is higher than 1100 °C, then the content of α-Al_2_O_3_ strongly increases; however, some other phases can be also present [7–9].

To prepare pure alumogel, liquid phase reactions are used with salt precursors such as Al(NO_3_)_3_, Al_2_(SO_4_)_3_, and Al(OH)_2_Cl [10–13]. Fast precipitation of the products of hydrolyzed salts of aluminum leads to formation of amorphous particles of alumogel [14]. Crystallization of alumogel in the aqueous media at room temperature occurs very slowly. In several months, crystallites of gibbsite (Al(OH)_3_) can form. Heating of alumogel at 98–100 °C gives boehmite (γ-AlO(OH)) in 1 h. Precipitated alumogel is composed of small globules (5–10 nm in diameter) and fiber-like particles. Needle-like particles (whiskers) formed during 1 h are of 10–20 nm in thickness.

Stable sol of boehmite can be prepared by peptization of precipitant formed upon mixing the solutions of Al(NO_3_)_3_ and NH_4_OH, and dried xerogel is macroporous [15, 16]. Another pathway of preparation of porous alumina using aluminum nitrate nonahydrate as a precursor is based on thermodestruction [17, 18]:

$$ \begin{array}{c}\hfill \mathrm{A}\mathrm{l}{\left({\mathrm{NO}}_3\right)}_3\cdot 9{\mathrm{H}}_2\mathrm{O}\kern0.5em \left(120-140{}^{\circ}\mathrm{C}\right)\ \to\ \mathrm{A}\mathrm{l}\left({\mathrm{NO}}_3\right){\left(\mathrm{O}\mathrm{H}\right)}_2\cdot 5{\mathrm{H}}_2\mathrm{O}\kern0.5em \left(150-180{}^{\circ}\mathrm{C}\right)\ \to \hfill \\ {}\hfill \mathrm{A}\mathrm{l}\left({\mathrm{NO}}_3\right){\left(\mathrm{O}\mathrm{H}\right)}_2,\kern0.5em {\mathrm{Al}}_2{\mathrm{O}}_3\cdot 3{\mathrm{H}}_2\mathrm{O},\kern0.5em {\mathrm{Al}}_2{\mathrm{O}}_3\cdot 2{\mathrm{H}}_2\mathrm{O},\kern0.5em {\mathrm{Al}}_2{\mathrm{O}}_3\cdot {\mathrm{H}}_2\mathrm{O}\left(200-250{}^{\circ}\mathrm{C}\right)\ \to \hfill \\ {}\hfill {\mathrm{Al}}_2{\mathrm{O}}_3\cdot {\mathrm{H}}_2\mathrm{O}\left(350-450{}^{\circ}\mathrm{C}\right)\to \mathrm{disperse}\kern0.5em {\mathrm{Al}}_2{\mathrm{O}}_3.\hfill \end{array} $$

Al(OH)_2_NO_3_∙H_2_O formed at 150–155 °C and partial pressure of water vapor of 120–130 kPa can form stable colloidal solution in aqueous medium. Impregnation of ceramics by this solution and then calcination at 550 °C results in the formation of coating with γ-Al_2_O_3_. Water content (water vapor) can influence the processes of thermodestruction of crystalline aluminum hydrates. Therefore, it is of interest to study thermal transformations of aluminum nitrate nonahydrate in an atmosphere free of water vapor. The aim of this work was to study these processes in a flow-type reactor with Ar atmosphere, as well as the structure, morphology, and texture of aluminas prepared at different temperatures of calcination of the precursor with α-Al_2_O_3_ as a final product.

## Methods

The characteristics of the products of thermodestruction of aluminum nitrate nonahydrate (ANN) were studied for samples treated at 350, 480, 850, and 1100 °C in the Ar atmosphere (Table 1, samples 1, 2, 3, and 4, respectively). Thermodecomposition of ANN was studied using a STA 449F3 Jupiter (Netzsch) thermoanalyzer. Thermogravimetry (TG) gives changes in the sample mass (errors <±1 mg), rate of changes in the mass (differential TG (DTG)), and enthalpy of the processes (differential thermal analysis (DTA), signal errors <±0.05 μW). ANN was heated from 20 to 300 °C at a heating rate *β* = 5 °C/min, and partially dehydrated ANN (Al(NO_3_)_3_∙7.5H_2_O) was heated from 20 to 1200 °C at *β* = 10 °C/min in the Ar flow at 30 ml/min.

**Table 1:** Conditions of preparation samples and their composition

Sample	Calcination temperature (°C)	Time of calcination (min)	Structural state	Phase composition
1	350	40	Amorphous	AlONO_3_·H_2_O (90 %)
				AlOOH (10 %)
2	480	40	Amorphous/crystalline	AlONO_3_·H_2_O (30 %)
				AlOOH (70 %)
3	850	40	Amorphous/crystalline	γ-Al_2_O_3_
4	1100	40	Crystalline	α-Al_2_O_3_

The textural characteristics of samples were analyzed on the basis of low-temperature (77.4 K) nitrogen adsorption-desorption isotherms recorded using a Quantachrome Autosorb Nova 2200c adsorption analyzer. Before measurements, the samples were heated at 180 °C for 24 h. The specific surface area (*S*_BET_) was calculated according to the standard Brunauer-Emmett-Teller (BET) method [19]. The total pore volume *V*_p_ was evaluated from the nitrogen adsorption at *p*/*p*_0_ ≈ 0.99, where *p* and *p*_0_ denote the equilibrium and saturation pressure of nitrogen at 77.4 K, respectively [20]. The nitrogen desorption data were used to compute the pore size distributions (PSDs, differential *f*_V_(*R*)~d*V*_p_/d*R* and *f*_S_(*R*)~d*S*/d*R*) using a self-consistent regularization (SCR) procedure under non-negativity condition (*f*_V_(*R*) ≥ 0 at any pore radius *R*) at a fixed regularization parameter *α* = 0.01 with a complex pore model with cylindrical (C) pores in alumina and voids (V) between spherical nonporous alumina nanoparticles packed in random aggregates (CV/SCR model) [21, 22]. The differential PSDs in respect to pore volume *f*_V_(*R*)~d*V*/d*R*, ∫*f*_V_(*R*)d*R*~*V*_p_ were re-calculated to incremental PSD (IPSD) at Φ_V_(*R*_*i*_) = (*f*_V_(*R*_*i*+1_) + *f*_V_(*R*_*i*_))(*R*_i+1_ − *R*_i_)/2 at ∑Φ_V_(*R*_*i*_) = *V*_p_). The differential *f*_S_(*R*) functions were used to estimate the deviation of the pore shape from the model $\varDelta w=\left({S}_{\mathrm{BET}}/{\displaystyle \underset{R_{\min }}{\overset{R_{\max }}{\int }}{f}_S(R)dR}\right)-1$, where *R*_max_ and *R*_min_ are the maximal and minimal pore radii, respectively [23]. The *f*_V_(*R*) and *f*_S_(*R*) functions were also used to calculate contributions of nanopores (*V*_nano_ and *S*_nano_ at 0.35 nm < *R* < 1 nm), mesopores (*V*_meso_ and *S*_meso_ at 1 nm < *R* < 25 nm), and macropores (*V*_macro_ and *S*_macro_ at 25 nm < *R* < 100 nm). Additionally, the PSD were calculated using nonlocal density functional theory (NLDFT) method [24] using equilibrium model with cylindrical pores.

X-ray diffraction (XRD) patterns of samples 1–4 were recorded over 2*θ* = 10°–65° range using a DRON–4–07 (Burevestnik, St. Petersburg) diffractometer with Cu K_α_(*λ* = 0.15418 nm) radiation and a Ni filter. A copper X-ray source may preferentially produce a beam of X-rays with wavelengths 0.154 and 0.139 nm. Nickel has an absorption edge at 0.149 nm, between the two copper lines. Thus, using nickel as a filter for copper would result in the absorption of the slightly higher energy 0.139 nm X-rays, while letting the 0.154 nm rays through without a significant decrease in intensity. Thus, a copper X-ray source with a Ni filter can produce a nearly monochromatic X-ray beam with photons of mostly 0.154 nm. Analysis of the crystalline structure of aluminas was carried out using the JCPDS Database (International Center for Diffraction Data, PA, 2001) [25].

The infrared (IR) spectra of powdered samples over the 4000–300 cm^−1^ range (at 4 cm^−1^ resolution) were recorded in transmittance mode using a Specord M80 (Carl Zeiss, Jena) spectrometer. Sample powders were stirred and pressed in a mixture with KBr (1:100, tables of ~20 mg). The spectra were re-calculated into the absorbance spectra.

A field emission-transmission electron microscope FE-TEM (JSM–2100F, Japan, voltage 200 kV) was used to record high-resolution TEM images of selected samples. A powder sample was added to acetone (for chromatography) and sonicated. Then, a drop of the suspension was deposited onto a copper grid with a thin carbon film. After acetone evaporation, sample particles remained on the film were studied with high-resolution transmission electron microscope (HRTEM).

## Results and Discussion

**Fig. 1:**
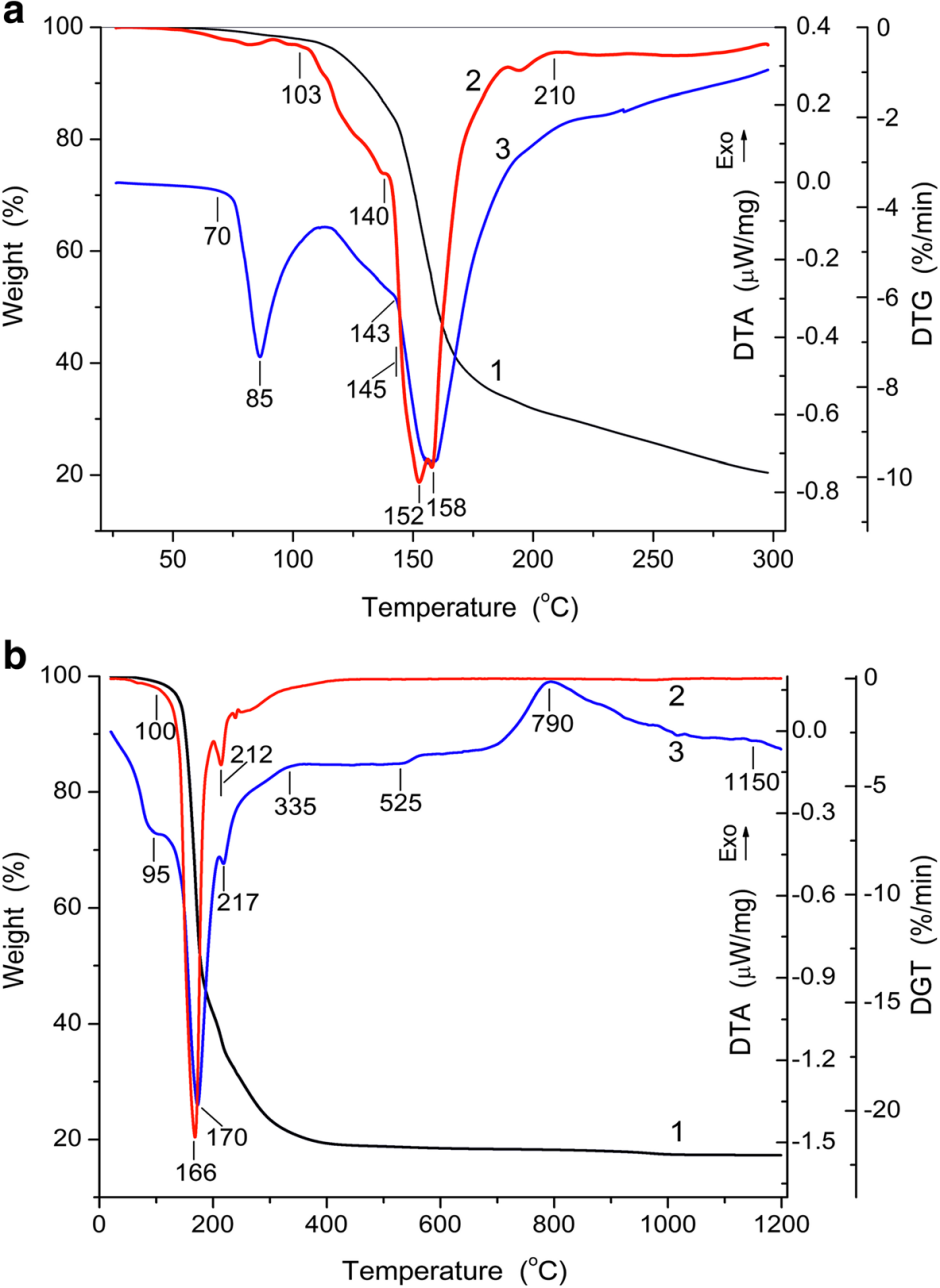
TG (curves 1), DTG (2), and DTA (3) curves upon heating of Al(NO_3_)_3_·9H_2_O to 300 °C at heating rate *β* = 5 °C/min (**a**) and Al(NO_3_)_3_·7.5H_2_O to 1200 °C at *β* = 10 °C/min (**b**)

Endo-effect (Fig. 1a, curve 3) in the range of 70–108 °C (maximum at 85 °C) shows melting of the aluminum nitrate nonahydrate with parallel diminution of the sample weight (by 14 wt.% at 140 °C) due to water desorption. This process can be described by reaction

1 $$ \mathrm{A}\mathrm{l}{\left({\mathrm{NO}}_3\right)}_3\cdot 9{\mathrm{H}}_2\mathrm{O}\to {\left[\mathrm{A}\mathrm{l}{\left({\mathrm{OH}}_2\right)}_6\right]}^{3+}\cdot 3{{\mathrm{NO}}_3}^{\hbox{--} }+3{\mathrm{H}}_2\mathrm{O} $$

with removal of 3H_2_O from ANN and formation of [Al(OH_2_)_6_]^3+^ bonding anions 3NO_3_^−^. In the range of 140–210 °C, the weight diminution is 38 wt.% (16.8 wt.% in the range of 140–152 °C) due to process with endo-maximum at 158 °C due to removal of water and acid and formation of oligomer structures

2 $$ {\left[\mathrm{A}\mathrm{l}{\left({\mathrm{OH}}_2\right)}_6\right]}^{3+}\cdot 3{{\mathrm{NO}}_3}^{\hbox{--}}\to {\left[\mathrm{A}\mathrm{l}\left(\mathrm{O}\mathrm{H}\right){\left({\mathrm{OH}}_2\right)}_5\right]}^{2+}\cdot 2{{\mathrm{NO}}_3}^{\hbox{--} }.+{\mathrm{HNO}}_3, $$

3 $$ n\left\{{\left[\mathrm{A}\mathrm{l}\left(\mathrm{O}\mathrm{H}\right){\left({\mathrm{OH}}_2\right)}_5\right]}^{2+}\cdot 2{{\mathrm{NO}}_3}^{\hbox{--}}\right\}\to\ {\left\{{\left[\mathrm{A}\mathrm{l}\mathrm{O}\left({\mathrm{OH}}_2\right)\right]}^{+}\cdot {{\mathrm{NO}}_3}^{\hbox{--}}\right\}}_n+n{\mathrm{H}\mathrm{NO}}_3+4n{\mathrm{H}}_2\mathrm{O}, $$

where *n* = 3 ÷ 30. In the oligomers, AlO_4_ structures can be formed.

To enhance the DTA sensitivity at high temperatures in the mentioned processes, the amounts of water in the initial sample can be reduced from Al(NO_3_)_3_·9H_2_O to Al(NO_3_)_3_·7.5H_2_O due to preheating at 120 °C for 1 h. The TG/DTG/DTA study of the preheated sample upon heating to 1200 °C at *β* = 10 °C/min (Fig. 1b) shows that the increase in the value of *β* leads to a typical shift of the extreme toward higher temperature.

A small extremum of DTG at 212 °C (Fig. 1b, curve 2) and DTA at 217 °C is due to the removal of the HNO_3_ molecules. In the 217–525 °C range, oligomers with [AlO(OH_2_)]^+^·NO_3_^−^ transform into aluminum hydroxide

4 $$ {\left[\mathrm{A}\mathrm{l}\mathrm{O}\left({\mathrm{OH}}_2\right)\right]}^{+}\cdot {{\mathrm{NO}}_3}^{\hbox{--} }=\mathrm{AlOOH}+{\mathrm{HNO}}_3. $$

The weight loss in this range is equal to 18 wt.%. The exo-effect (maximum at 790 °C) is due to transformation of aluminum hydroxide into oxide γ-Al_2_O_3_ at 525–1000 °C (see Additional file 1: Figure S1)

5 $$ 2\mathrm{AlOOH}={\mathrm{Al}}_2{\mathrm{O}}_3+{\mathrm{H}}_2\mathrm{O}. $$

The endo-effect at *T* > 1100 °C is due to transformation of γ-Al_2_O_3_ into α-Al_2_O_3_ (Fig. 1b and in the Additional file 1: Figure S1).

**Fig. 2:**
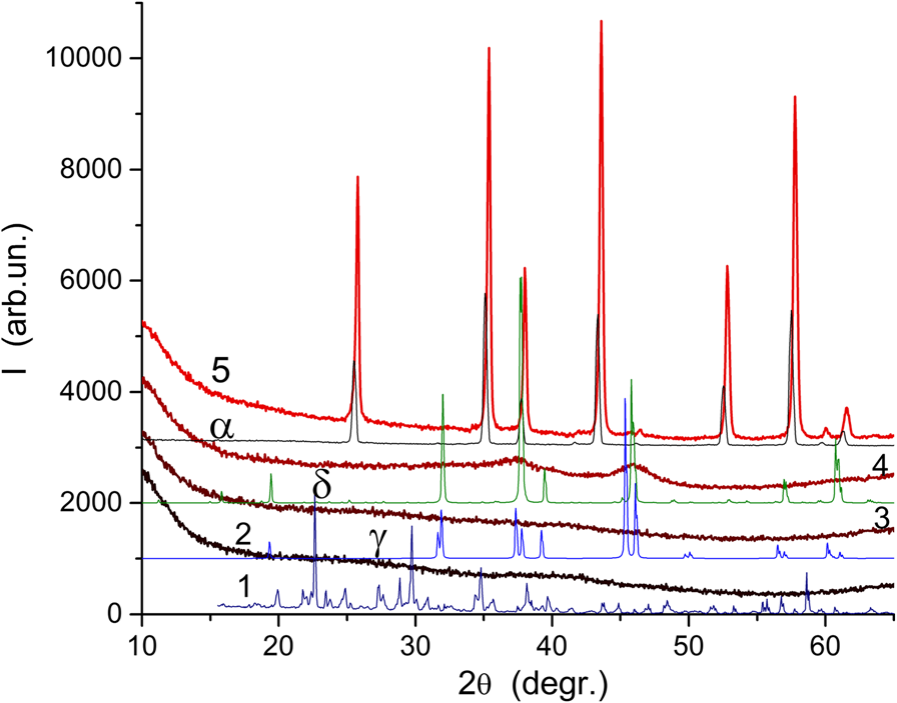
XRD patterns of Al(NO_3_)_3_·9H_2_O (curve *1*) and samples heated at 350 °С (*2*), 480 °С (*3*), 850 °С (*4*), and 1100 °С (*5*) and pure crystalline *γ*-, *δ*-, and *α*-Al_2_O_3_

**Fig. 3:**
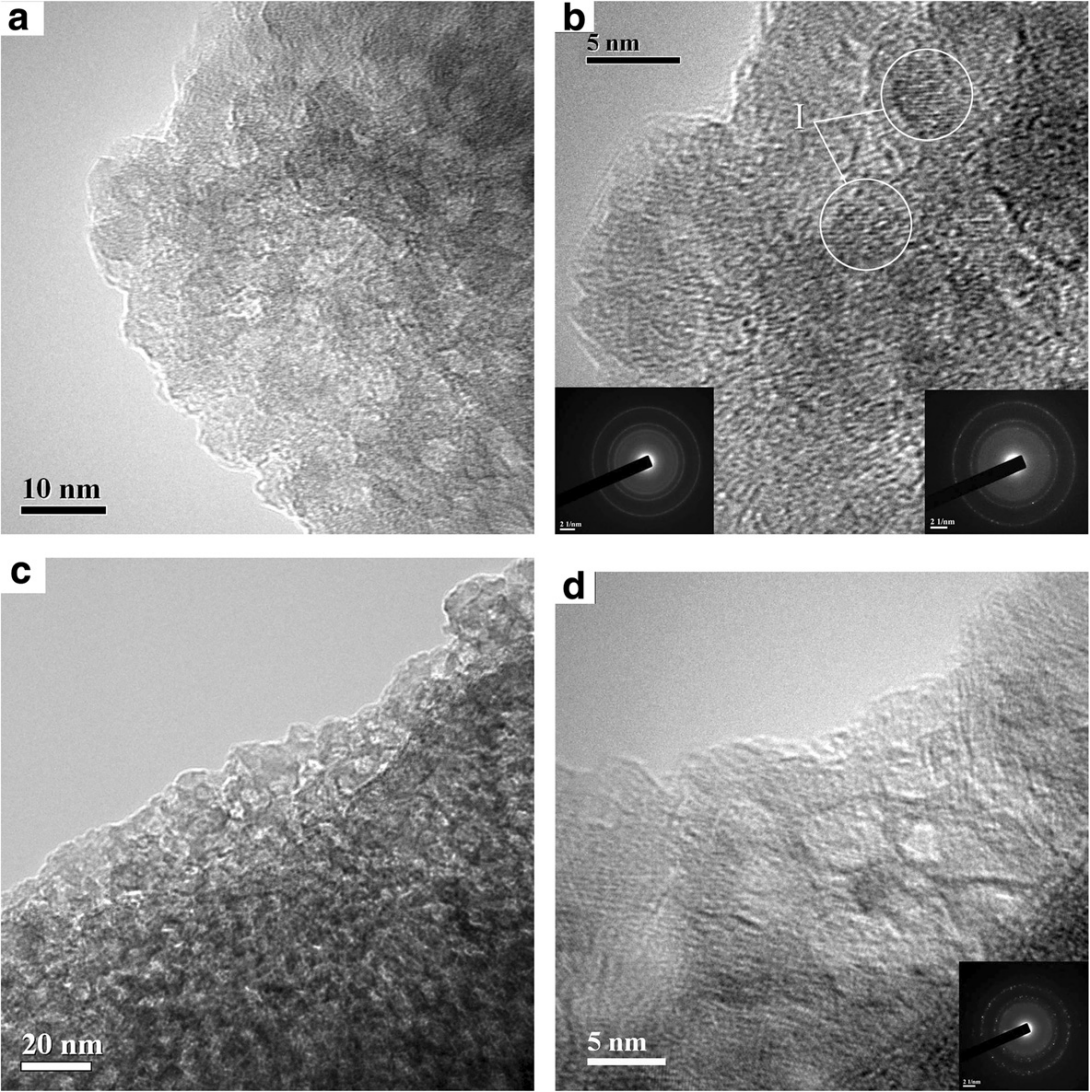
HRTEM images of samples 2 (**a**, **b**) and 3 (**c**, **d**) at different scales

Changes in the phase composition of samples heated at different temperatures were studied using the XRD method (Fig. 2). The XRD pattern of sample 3 (Fig. 2, curve 4) shows appearance of crystalline nanoparticles and certain contribution of XRD amorphous alumina. According to the XRD patterns of pure crystalline aluminas (Fig. 2) and temperature ranges of stabilization of various aluminas (see Additional file 1: Figure S1), one can assume that the crystalline structures (formed upon heating at 850 °C) correspond mainly to γ-Al_2_O_3_ nanoparticles of 5–8 nm in size (Fig. 3c, d). A small size of them causes broadening of the XRD lines (Fig. 2). The lattice constant *a* = 0.79431 nm for sample 3 is larger than that for bulk γ-Al_2_O_3_ (see Additional file 1: Table S1). This effect is a consequence of nano-scaled size of crystallites with certain distortions in the surface layers of nanocrystallites (due to effects of the Laplace pressure and surface tension with decreasing particle size and increasing surface curvature) with great contribution of these layers into the volume of nanoparticles. Sample 4 prepared by calcination of ANN at 1100 °C is composed of α-Al_2_O_3_ crystallites (Fig. 2, curve 5) at lattice constants *a* = *b* = 0.47579(5) ± 0.00005(3) nm and *c* = 1.29890(7) ± 0.00012(7) nm.

Samples with ANN heated at 350 °C (Fig. 2, curve 2, AlONO_3_·H_2_O) and 480 °C (curve 3, AlOOH) are XRD amorphous. HRTEM images of sample 2 preheated at 480 °C (Fig. 3a, b) show nanoglobules of 6–10 nm in size, which are mainly amorphous and composed of the Al–O chains of 1–5 nm in length. However, some nanoparticles demonstrate crystalline structure (see Fig. 3b, marked structures).

**Fig. 4:**
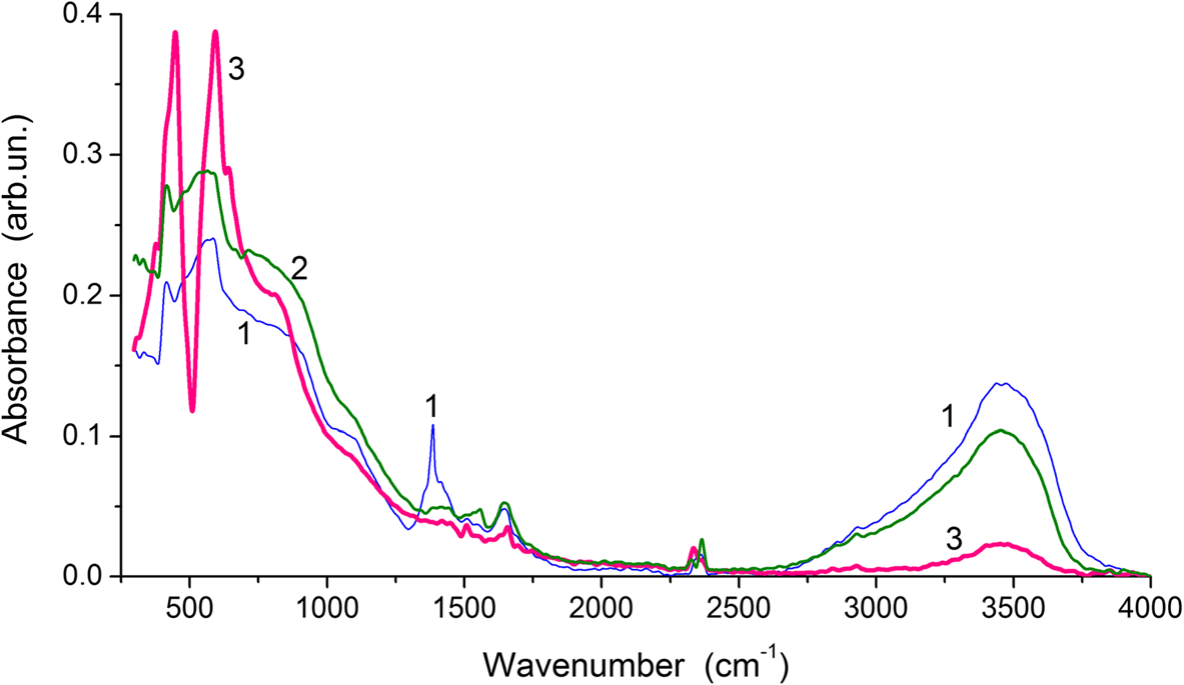
IR spectra of samples 2 (curve *1*), 3 (*2*), and 4 (3)

The presence of oligomer (with unit AlONO_3_·H_2_O) chains with the Al–O bonds is confirmed by the IR spectra (Fig. 4). A band at 1385 cm^−1^ in the IR spectrum of sample 2 (Fig. 4, curve 1) is due to stretching vibrations of NO_3_^−^ [26–28], and this band is absent in samples 3 and 4 undergoing heating at higher temperatures with removal nitro groups, i.e., pure alumina is formed.

The presence of both tetrahedral AlO_4_ and octahedral AlO_6_ structures in aluminas results in broadening of the Al–O stretching and deformation vibration bands including transverse (TO) and longitudinal (LO) optical components. The broad bands at 1100–650 cm^−1^ and 650–300 cm^−1^ can be roughly assigned to tetrahedral and octahedral structures in aluminas, respectively, with contributions of asymmetric (larger wavenumbers *ν*) and symmetric (smaller *ν*) vibrations. Corundum is mainly composed of octahedral AlO_6_ structures with a small amount of AlO_4_ in the surface layers and amorphous fragments. Sample 4 with corundum is the most ordered among all studied samples (Fig. 2). Therefore, the bands related to the Al–O stretching vibrations are narrower at *ν* < 700 cm^−1^ linked to the AlO_6_ structures (Fig. 4, curve 3) than those of samples 2 (curve 1) and 3 (curve 2). Additionally, relative contributions of bands at *ν* > 700 cm^−1^ (linked to AlO_4_ structures) decrease for sample 4.

Water is observed in all samples (a broad band of the O–H stretching vibrations at 3800–3000 cm^−1^ and deformation vibrations at 1660–1650 cm^−1^). However, its content decreases in a line of samples AlOOH (sample 2) > γ-Al_2_O_3_ (3) > α-Al_2_O_3_ (4) because of a decrease in the specific surface area (Table 2, *S*_BET_) and pore volume (*V*_p_) resulting in diminution of water adsorption under the same conditions [9]. Weak bands at 1552–1514 cm^−1^ can be attributed to HCO_3_^−^ and CO_3_^2−^ [29].

**Table 2:** Textural characteristics of samples heated at 350 °С (1), 480 °С (2), 850 °С (3), and 1100 °С (4)

Sample	*S*_BET_(m^2^/g)	*S*_nano_(m^2^/g)	*S*_meso_(m^2^/g)	*S*_macro_(m^2^/g)	*V*_p_(cm^3^/g)	*V*_nano_(cm^3^/g)	*V*_meso_(cm^3^/g)	*V*_macro_(cm^3^/g)	Δ*w*	<*R*_V_> (nm)	<*R*_S_> (nm)	*c*_void_	*c*_cyl_
1	66	29	37	0	0.044	0.009	0.035	0	0.153	1.94	1.28	0.392	0.608
2	180	34	146	0	0.138	0.007	0.131	0	0.470	1.97	1.65	0.697	0.303
3	77	6	71	0	0.086	0.001	0.080	0.005	0.397	8.14	2.44	0.536	0.464
4	14	3	9	2	0.070	0.001	0.032	0.037	0.292	33.55	10.39	0.538	0.462

Note. $<{R}_V>={\displaystyle \underset{R_{\min }}{\overset{R_{\max }}{\int }}R{f}_V(R)dR}/{\displaystyle \underset{R_{\min }}{\overset{R_{\max }}{\int }}{f}_V(R)dR}$; $<{R}_S>={\displaystyle \underset{R_{\min }}{\overset{R_{\max }}{\int }}R{f}_S(R)dR}/{\displaystyle \underset{R_{\min }}{\overset{R_{\max }}{\int }}{f}_S(R)dR}$; *c*_void_ and *c*_cyl_ are the weight coefficients corresponding to contributions of voids between spherical particles and cylindrical pores in CV/SCR

**Fig. 5:**
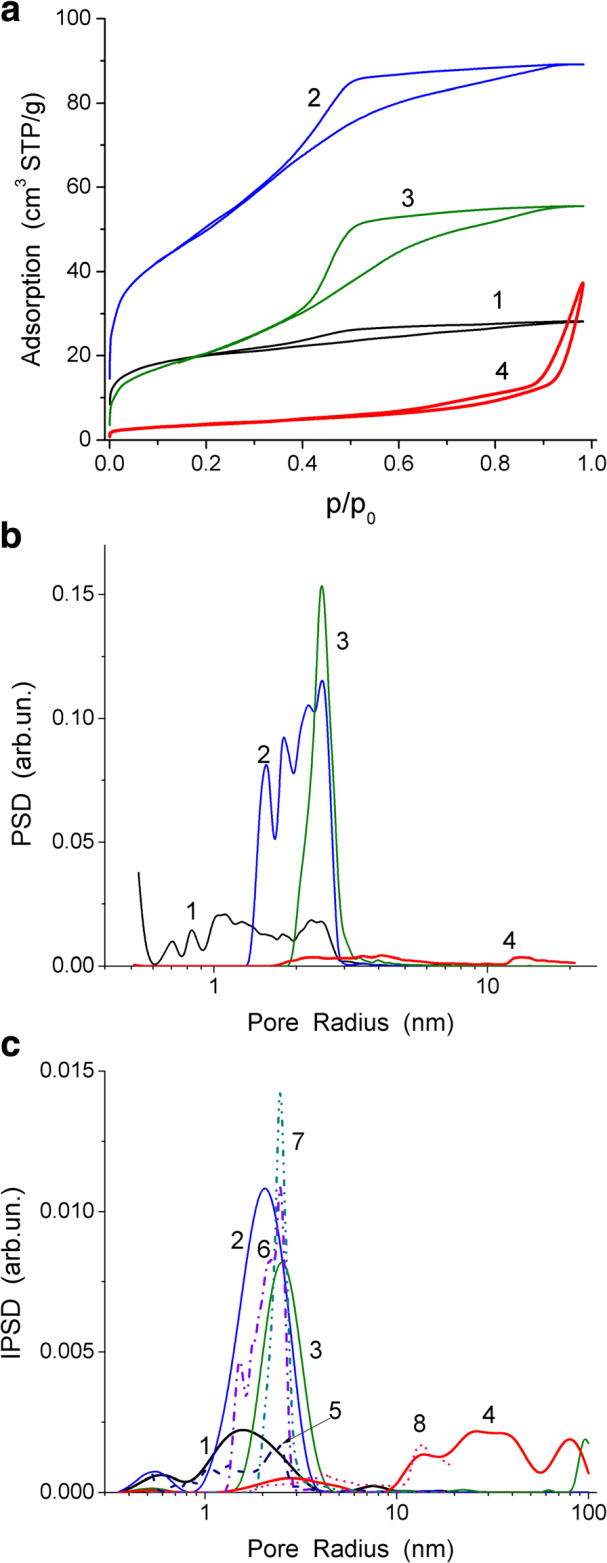
**a** Nitrogen adsorption-desorption isotherms, **b** NLDFT differential, and **c** incremental CV/SCR pore size distributions (*solid lines*) for samples 1 (curves 1), 2 (2), 3 (3), 4 (4), and **c** incremental NLDFT PSD (*dotted*-*dashed lines*) for samples 1 (curve *5*), 2 (*6*), 3 (*7*), 4 (*8*)

The textural characteristics of samples 1–4 (Table 2, Fig. 5) depend strongly on calcination temperature. Maximal porosity (Table 2, *V*_p_) and specific surface area (*S*_BET_) are characteristics for sample 2 calcined at *T*_t_ = 480 °C. Subsequent increase in the value of *T*_t_ results in a significant decrease in the porosity, especially for sample 4 treated at *T*_t_ = 1100 °C. Samples 1–3 have mainly narrow mesopores (Table 2, *S*_meso_, *V*_meso_, Fig. 5b, c) at small contribution of nanopores (*S*_nano_, *V*_nano_). Sample 4 possesses broad PSD (Fig. 5b, c) with increased contribution of macropores (*S*_macro_, *V*_macro_). Broadening of pores of samples with increasing calcination temperature is well observed in changes in the average pore radius in respect to pore volume (Table 2, <*R*_V_>) and specific surface area (<*R*_S_>). These changes can be explained by two effects. First, nanoglobules become smaller upon heating at higher temperatures due to removal of water, acid HNO_3_, or anions NO_3_^−^. Second, nanoparticles more strongly fuse with increasing temperature. The shape of pores is complex since the values of Δ*w* (Table 2) characterizing the deviation of the pore model are large. Note that in the case of silica gels, the value of Δ*w* can be much smaller up to Δ*w* ≈ 0.01 calculated using simple cylindrical pore model due to quite cylindrical shape of pores [9] in contrast to materials studied here (Fig. 2).

The investigation of phase-formation processes during thermolytic destruction of aluminum nitrate nonahydrate reveals new aspects of the synthesis of aluminum hydroxid-oxide and aluminum oxide compounds. Materials obtained in such way are nanostructured and chemically pure. They have specific physicochemical properties and may be used as adsorbents and catalyst carriers. It is possible to obtain single crystals of sapphire and ruby on its basis.

The complex water-soluble compound [AlOH(OH)_2_]^2+^·2NO_3+_^−^, formed in this process, was the convenient precursor to form secondary mesoporous Al_2_O_3_-structure in silica-alumina ceramics by impregnation and calcination methods. Ceramics may be used as a membrane for ultrafiltration water purification after correction of the porous structure. This water-soluble compound is also used to form an active aluminum oxide layer on the surface of the cellular cordierite ceramic that is a carrier for the platinum-palladium catalyst.

In addition, nanostructured materials possess the properties strongly different from those of bulk materials, and this difference depends on the morphology of nanomaterials. Therefore, there is a fundamental problem to control the morphology of nanoparticulate materials, which tend to aggregate.

## Conclusions

Investigations of calcination effects on the transformations of aluminum nitrate nonahydrate into boehmite, γ-Al_2_O_3_, and α-Al_2_O_3_ show that it occurs according to scheme

$$ \begin{array}{c}\hfill \mathrm{A}\mathrm{l}{\left({\mathrm{NO}}_3\right)}_3\cdot 9{\mathrm{H}}_2\mathrm{O}\ \left(100-140{}^{\circ}\mathrm{C}\right)\to {\left[\mathrm{A}\mathrm{l}{\left({\mathrm{O}\mathrm{H}}_2\right)}_6\right]}^{3+}\cdot 3{{\mathrm{NO}}_3}^{\hbox{--}}\left(140-152{}^{\circ}\mathrm{C}\right)\to \hfill \\ {}\hfill {\left[\mathrm{Al}\mathrm{OH}{\left({\mathrm{O}\mathrm{H}}_2\right)}_5\right]}^{2+}\cdot 2{{\mathrm{NO}}_3}^{\hbox{--}}\left(152-210{}^{\circ}\mathrm{C}\right)\to {\mathrm{Al}\mathrm{ONO}}_3\cdot {\mathrm{H}}_2\mathrm{O}\left(210-525{}^{\circ}\mathrm{C}\right)\to \hfill \\ {}\hfill \mathrm{Al}\mathrm{OOH}\left(525-1000{}^{\circ}\mathrm{C}\right)\to \upgamma \hbox{-} {\mathrm{Al}}_2{\mathrm{O}}_3\left(1100{}^{\circ}\mathrm{C}\right)\to \upalpha \hbox{-} {\mathrm{Al}}_2{\mathrm{O}}_3.\hfill \end{array} $$

Amorphous and crystalline phases of boehmite formed at *T* > 210 °C represent nanoparticles of 6–10 nm in size strongly aggregated since *S*_BET_ = 66 m^2^/g is smaller than it should be for unbound nanoparticles of the same sizes. In the amorphous phase, chains with –AlOH–O–AlOH– have the length of 1–5 nm. Heating at 350–525 °C results in the formation of mesoporous aluminum hydroxide with decreasing size (2.5–5.0 nm) of nanoparticles. However, tight joining of these nanoglobules strongly reduces the specific surface area to 180 m^2^/g instead of 350 m^2^/g for individual spherical nanoparticles of similar sizes. Subsequent heating at 850 °C leads to enhanced binding of γ-Al_2_O_3_ nanoparticles in strongly aggregated structures, and *S*_BET_ drops down to 77 m^2^/g. Corundum particles formed at 1100 °C are characterized by additionally increased joining in the aggregates because the value of *S*_BET_ is minimal (14 m^2^/g).

## Additional File

Additional file 1: Figure S1 and Table S1.
**Figure S1.** Thermal transformation sequence of the aluminum hydroxides: gibbsite Al(OH)_3_, bayerite α-Al(OH)_3_ and β-Al(OH)_3_, boehmite γ-AlO(OH), and diaspore α-AlO(OH) into different phases χ, ρ, η, γ, δ, θ, and α-alumina characterized by different ranges of thermal stability. **Table S1.** Structural parameters of crystalline aluminas. (PDF 150 kb)

## Abbreviations

ANN: aluminum nitrate nonahydrate
BET: Brunauer-Emmett-Teller
DTA: differential thermal analysis
DTG: differential thermogravimetry
HRTEM: high-resolution transmission electron microscope
IR: infrared
NLDFT: nonlocal density functional theory
PSD: pore size distribution
SCR: self-consistent regularization
TG: thermogravimetry
XRD: X-ray diffraction
