# Supplementary material for: Structural and Morphological Features of Disperse Alumina Synthesized Using Aluminum Nitrate Nonahydrate
Source: Nanoscale Res Lett. 2016 Mar 22;11:153. doi: 10.1186/s11671-016-1366-0 (PMC4801842; doi:10.1186/s11671-016-1366-0)
Supplement: Additional file 1: Figure S1 and Table S1. — Figure S1. Thermal transformation sequence of the aluminum hydroxides: gibbsite Al(OH)3, bayerite α-Al(OH)3 and β-Al(OH)3, boehmite γ-AlO(OH), and diaspore α-AlO(OH) into different phases χ, ρ, η, γ, δ, θ, and α-alumina characterized by different ranges of thermal stability. Table S1. Structural parameters of crystalline aluminas. (PDF 150 kb) [file 11671_2016_1366_MOESM1_ESM.pdf]

Fig. S1 and Table S1 show structural features vs. temperature for a variety of hydroxides and oxides of alumina.

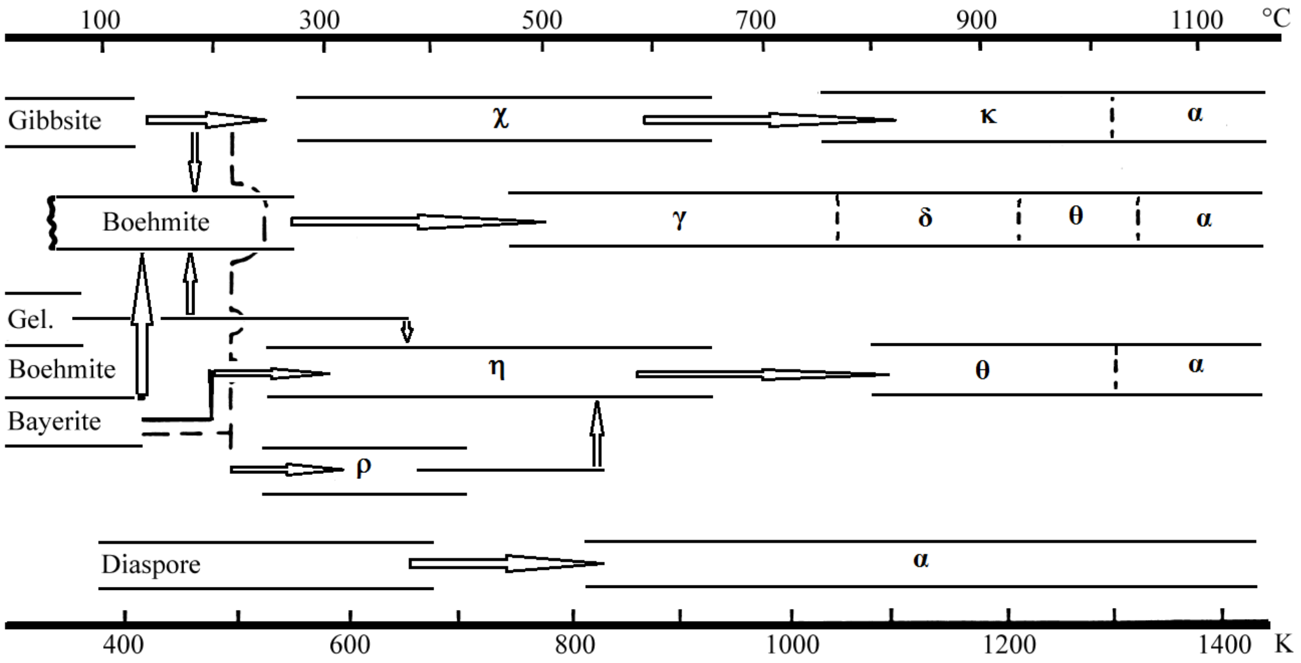

Fig. S1. Thermal transformation sequence of the aluminum hydroxides: gibbsite $Al(OH)_3$, **bayerite** α-$Al(OH)_3$ and β-$Al(OH)_3$, boehmite γ-AlO(OH), and diaspore α-AlO(OH) into different phases χ, ρ, η, γ, δ, θ, and α-alumina characterized by different ranges of thermal stability [1,2].

Table S1 Structural parameters of crystalline aluminas

Alumina	Space group of symmetry	Lattice constants (Å)	Angle (derg.)	Density (g/cm^3)	References
α-Al_2O_3	R-3cH	4.7640(1)<br>4.7640(1)<br>13.0091(3)	90.0<br>90.0<br>120.0	3.97	[3]
θ-Al_2O_3	C12/m1	11.795(5)<br>2.91(1)<br>5.621(7)	90.0<br>103.79<br>90.0	3.61	[4]
χ-Al_2O_3	Pna21	4.8437(2)<br>8.3300(3)<br>8.9547(4)	90.0<br>90.0<br>90.0	3.75	[5]
η-Al_2O_3	Fd-3mS	7.906<br>7.906<br>7.906	90.0<br>90.0<br>90.0	3.65	[6]
γ-Al_2O_3	Fd-3mZ	7.887<br>7.887<br>7.887	90.0<br>90.0<br>90.0	3.68	[7]
σ-Al_2O_3	Fd-3mS	7.948(2)<br>7.948(2)<br>7.948(2)	90.0<br>90.0<br>90.0	3.60	[8]
δ-Al_2O_3	P-4m2	5.599(10)<br>5.599(10)<br>23.657(50)	90.0<br>90.0<br>90.0	3.65	[9]

References

[1] Wefers K, Misra C. Oxides and Hydroxides of Aluminum. Pennsilvania: ALCOA, Laboratories; 1987.

[2] Souza Santos P, Souza Santos H, Toledo SP. Standard transition aluminas. Electron microscopy studies. Materials Research. 2000;3:104-14.

[3] Cox DE, Moodenbaugh AR, Sleight AW, Chen H-Y. Structural refinement of neutron and X-ray data by the rietveld method: application to Al_2O_3 and $BiVO_4$. National Bureau of Standards (U.S.), Special Publication. 1980;567:189-201.

[4] Husson E, Repelin Y. Structural studies of transition aluminas. Theta alumina. European Journal of Solid State Inorganic Chemistry. 1996;33:1223-31.

[5] Ollivier B, Retoux R, Lacorre P, Massiot D, Ferey G. Crystal structure of kappa-alumina: an X-ray powder diffraction, TEM and NMR study. J Mater Chem. 1997;7:1049-56.

[6] Shirasuka K, Yanagida H., Yamaguchi G. The preparation of eta alumina and its structure. Journal of the Ceramic Association of Japan. 1976;84:610-3.

[7] Gutierrez G, Taga A, Johansson B. Theoretical structure determination of gamma-(Al_2O_3). Phys Rev Serie 3. B - Condensed Matter. 2001;65:0121011-4.

[8] Guse W, Saalfeld H. X-ray characterization and structure refinement of a new cubic alumina phase (sigma-Al_2O_3) with spinel-type structure. Neues Jahrbuch fuer Mineralogie. Monatshefte. 1990;5:217-26.

[9] Repelin Y, Husson E. Etudes structurales d'alumines de transition. I - Alumines gamma et delta Materials. Research Bulletin. 1990;25:611-21.
